# School-based group interpersonal therapy for adolescents with depression in rural Nepal: a mixed methods study exploring feasibility, acceptability, and cost

**DOI:** 10.1017/gmh.2022.46

**Published:** 2022-08-22

**Authors:** Kelly Rose-Clarke, Prakash B. K., Jananee Magar, Indira Pradhan, Pragya Shrestha, Eliz Hassan, Gerard J. Abou Jaoude, Hassan Haghparast-Bidgoli, Delan Devakumar, Ludovico Carrino, Ginevra Floridi, Brandon A. Kohrt, Helen Verdeli, Kathleen Clougherty, Alexandra Klein Rafaeli, Mark Jordans, Nagendra P. Luitel

**Affiliations:** 1 Department of Global Health and Social Medicine, King's College London, London, UK; 2 Transcultural Psychosocial Organization Nepal, Kathmandu, Nepal; 3 Institute for Global Health, University College London, London, UK; 4 Department of Economics, Business, Mathematics and Statistics, University of Trieste, Trieste, Italy; 5 Leverhulme Centre for Demographic Science, University of Oxford, Oxford, UK; 6 Department of Psychiatry, George Washington University, Washington, DC, USA; 7 Teachers College, Columbia University, New York, NY, USA; 8 Psychological Services, Ruth and Allen Ziegler Student Services, Tel Aviv University, Tel Aviv, Israel; 9 Centre for Global Mental Health, King's College London, London, UK

**Keywords:** Adolescent, depression, interpersonal therapy, Nepal

## Abstract

**Background:**

Adolescents with depression need access to culturally relevant psychological treatment. In many low- and middle-income countries treatments are only accessible to a minority. We adapted group interpersonal therapy (IPT) for adolescents to be delivered through schools in Nepal. Here we report IPT's feasibility, acceptability, and cost.

**Methods:**

We recruited 32 boys and 30 girls (aged 13–19) who screened positive for depression. IPT comprised of two individual and 12 group sessions facilitated by nurses or lay workers. Using a pre-post design we assessed adolescents at baseline, post-treatment (0–2 weeks after IPT), and follow-up (8–10 weeks after IPT). We measured depressive symptoms with the Depression Self-Rating Scale (DSRS), and functional impairment with a local tool. To assess intervention fidelity supervisors rated facilitators' IPT skills across 27/90 sessions using a standardised checklist. We conducted qualitative interviews with 16 adolescents and six facilitators post-intervention, and an activity-based cost analysis from the provider perspective.

**Results:**

Adolescents attended 82.3% (standard deviation 18.9) of group sessions. All were followed up. Depression and functional impairment improved between baseline and follow-up: DSRS score decreased by 81% (95% confidence interval 70–95); functional impairment decreased by 288% (249–351). In total, 95.3% of facilitator IPT skills were rated superior/satisfactory. Adolescents found the intervention useful and acceptable, although some had concerns about privacy in schools. The estimate of intervention unit cost was US $96.9 with facilitators operating at capacity.

**Conclusions:**

School-based group IPT is feasible and acceptable in Nepal. Findings support progression to a randomised controlled trial to assess effectiveness and cost-effectiveness.

## Introduction

Improving the mental health of adolescents aged 10–19 is a global priority. Estimates suggest that 13% of adolescents are living with a mental disorder, equating to around 166 million worldwide (UNICEF, 2021). Poor mental health in adolescence can impair physical health, socio-emotional development, and educational attainment, limiting opportunities to thrive in adulthood (Thapar *et al*., 2012). Moreover, mental disorders are causally linked to suicide which is the fifth most prevalent cause of adolescent death (UNICEF, 2021).

Depression is a common mental disorder which accounts for 11% of total years lived with disability among adolescents aged 15–19 and 7% among adolescents aged 10–14 (Mokdad *et al*., 2016). The burden is highest in low- and middle-income countries (LMICs), yet the availability of mental health care in these settings is especially low (Patel *et al*., 2018). In an effort to overcome this treatment gap, the World Health Organization (WHO) recommends evidence-based psychological interventions for treating depression in adolescents (World Health Organization, 2016). Some of these interventions were developed in high-income countries and exporting them to LMIC settings poses multiple challenges (Benish *et al*., 2011; Hall *et al*., 2016; Cuijpers *et al*., 2018). Mental health care in LMICs tends to be institutionalised and trained mental health personnel at the community level are scarce. Primary health care systems are often under-resourced and overburdened, with limited mental health care capacity. Cultural challenges relate to divergent local concepts of distress and community perceptions of the acceptability and utility of mental health diagnoses and care (Heim and Kohrt, 2019, Sangraula *et al*., 2021).

The past decade has seen a rise in randomised controlled trials (RCTs) of psychological treatments adapted for LMICs, but the evidence base for treatments in children and adolescents is trailing that for adults. Recent meta-analyses demonstrated that effect sizes of psychological treatments are smaller in children than in adults (Cuijpers *et al*., 2020). An umbrella review of psychological interventions in humanitarian settings found adequate evidence to support treatments for children with conduct problems and post-traumatic stress disorder (PTSD), but not depression (Barbui *et al*., 2020). Research that tests psychological interventions for children and adolescents with depression is needed to build the evidence base in LMICs.

Interpersonal therapy (IPT) is one such psychological intervention that was developed in the USA to treat depression among adults and has been successfully adapted for adolescents in LMIC settings (Mufson *et al*., 2004*a*, 2004*b*; Bolton *et al*., 2007; Rosselló *et al*., 2008; O'Shea *et al*., 2015). The therapeutic focus of IPT is on the interpersonal context of depression, codified into key interpersonal triggers of depressive episodes, the four ‘interpersonal problem areas’: grief, disputes, role transitions, and social isolation. The persons are helped therapeutically by understanding the connection between their depression and the problem areas associated with it, and by building skills to manage these problems more effectively. Meta-analyses suggest IPT reduces depressive symptoms in adolescents (*d* = −1.48) and may be more acceptable to them than other psychological therapies (Zhou *et al*., 2015; Duffy *et al*., 2019; Zhou *et al*., 2020).

Nepal is a lower middle-income country where psychological intervention for depression in adolescents is urgently needed. The earthquakes in 2015 compounded effects of a decade-long war on the stability and prosperity of Nepali society and the economy. Over 8000 people were killed, over 16 000 injured, and many more left homeless or displaced (Ministry of Home Affairs, 2015). Monsoon rains, flooding, and landslides continue to drive people from their homes. After the earthquakes, the prevalence of adolescent depression was 40% in some areas (Silwal *et al*., 2018). Violence at home and in school, poverty, and parental migration are key risk factors for depression among Nepali adolescents. The COVID-19 pandemic and national lockdown pose further risks to their mental health (Orben *et al*., 2020; Kola *et al*., 2021). Existing mental health services are concentrated in urban centres and specialised services for children and adolescents are virtually non-existent (Luitel *et al*., 2015).

In this study we aimed to evaluate the feasibility of IPT for depressed adolescents in Nepal. We chose IPT because it is potentially compatible with Nepali conceptualisations of identity and distress, can be delivered by non-specialist workers, and can be conducted in groups which is likely to be more cost-effective and culturally acceptable than one-to-one therapy (Tol *et al*., 2005; Rose-Clarke *et al*., 2021). We adapted IPT for delivery in schools based on adolescents' and parents' preferences, and evidence that school-based psychological interventions are effective, scalable, and sustainable (Michelson *et al*., 2020, Clarke *et al*., 2021).

## Methods

We conducted a mixed-methods, uncontrolled pre-post evaluation to explore the feasibility of IPT and inform decisions to progress to an RCT. We use *feasibility* as an umbrella term that includes acceptability, utility, fidelity, outcome trends, and cost (Eldridge *et al*., 2016).

### Setting

Four government secondary schools in Sindhupalchowk took part. Sindhupalchowk is a mountainous district on the Nepal–Tibet border. The population (c. 288 000) mainly lives in rural areas and agriculture is the main source of income (UN Women, 2016). Life expectancy is 62 years. Net enrolment rates for lower secondary and secondary level schools are 78 and 43% respectively (UN Women, 2016).

### Intervention

We culturally and developmentally adapted the WHO group IPT intervention for delivery in schools in Nepal. Intervention components and adaptations made are detailed elsewhere (Rose-Clarke *et al*., 2020) and included: (i) replacing the term *depression* with *heart-mind problems* (*manko samasya*), which is a non-stigmatising Nepali term (Kohrt and Harper, 2008); (ii) framing the intervention as life skills training to mitigate stigma towards participants; and (iii) including singing, dancing, and storytelling to help build relationships within groups and improve engagement. We included two pre-group sessions: the first with the adolescent at school to identify the most relevant IPT problem areas, help the adolescent link their depressive symptoms to the problem area, and gather information about their interpersonal relationships and history of depression; the second with the adolescent and their parent, ideally at home, to mobilise parental support and build rapport with the adolescent's family. Adolescents and parents initially provided consent to participate in the first pre-group session (adolescents took an information sheet and consent form home from school for signing). The IPT facilitator collected consent for the group sessions in the second pre-group session. Adolescents were then invited to 12 weekly group sessions. Groups were gender specific. The initial session focused on encouraging adolescents to review and share their problems, and instilling hope for recovery. In middle sessions (2–11) adolescents practiced new interpersonal skills and offered and received support to resolve their problems. In the last session, they reviewed and celebrated their progress, and made plans to tackle future problems.

In each session adolescents reviewed their depressive symptoms using the Patient Health Questionnaire – Adolescent Version (PHQ-A) (Johnson *et al*., 2002). This was an integral part of IPT to help adolescents link changes in their symptoms to events in their daily lives. It also enabled facilitators to identify deterioration and suicidality. We developed a standard operating procedure to manage adolescents reporting suicidal thoughts, which included risk assessment, consultation with an IPT supervisor, communication with parents, and one-to-one intervention with a psychosocial counsellor.

We initially recruited and trained nine IPT facilitators. We recruited staff nurses because of a national policy to appoint nurses in secondary schools across the country. However, most nurses in Nepal are female and in the formative research adolescents said they preferred facilitators of the same gender, so we also recruited community lay facilitators. Facilitator training involved: (i) a 10-day in-person training to build basic psychosocial skills, including interpersonal communication, listening, non-verbal communication, and group management skills; (ii) a didactic 10-day workshop using the IPT manual; (iii) an IPT knowledge test; and (iv) practice using IPT skills with small groups of adolescents. Supervisors assessed facilitators' competency using the ENhancing Assessment of Common Therapeutic factors (ENACT) rating scale and the Working with children – Assessment of Competencies Tool (WeACT) (Kohrt *et al*., 2015; Jordans *et al*., 2021). Supervisors also used standardised lists to rate IPT-specific tasks carried out by facilitators in the sessions. From the nine facilitators we selected six (two nurses and four lay workers) based on their competency and availability, ensuring an equal number of male and female facilitators.

Facilitators worked in pairs. Six out of eight groups were facilitated by a nurse-lay worker pair. Group sessions took place between December 2019 and April 2020, in a classroom, library, or outside the school if space was unavailable. We ran eight groups (four female, four male) of five to nine adolescents. Facilitators were supervised by Nepali IPT supervisors (IP and PS). Supervisors were trained and supervised themselves by IPT master trainers (HV, KC, and AKR).

### Participants

IPT participants were 62 adolescents (32 boys and 30 girls) with depression and functional impairment. We used a two-stage process to recruit adolescents from four mixed gender, government secondary schools. First, we used the Community Informant Detection Tool (CIDT) adapted for adolescents to identify those with depression (Jordans *et al*., 2015). The tool comprised of a vignette and illustrations of adolescent depression. In schools, researchers discussed the vignette with adolescents and asked those who felt they were experiencing similar symptoms to complete the CIDT. This involved the adolescent matching symptoms presented in the vignette with their own symptoms and evaluating the extent to which symptoms compromised daily functioning. Second, we invited probable cases for further screening. Inclusion criteria were: adolescents aged 13–19; score of 14 or more on the Depression Self Rating Scale (DSRS); and score of four or more on a measure of functional impairment (Jordans *et al*., 2010; Kohrt *et al*., 2011). Exclusion criteria were current suicidal ideation with a current plan or a plan in the last 3 months; suicide attempt in the past 3 months; alcohol or substance abuse; and inability to participate in interviews due to severe cognitive or physical impairment, including severe mental disorders such as bipolar disorder or schizophrenia.

### Quantitative measures of feasibility

We calculated the participation rate as the number of adolescents who participated in group sessions as a percentage of all those eligible. We calculated the mean percentage attendance at group sessions (attendance at pre-group sessions was a prerequisite for receiving the intervention). IPT supervisors observed 27 of 90 group sessions. For each group, they aimed to observe at least one session from initial, middle, and termination phases. During these in-person observations supervisors assessed intervention fidelity using standardised lists to rate how well facilitators carried out key components of the session. Lists were adapted from tools in the WHO IPT manual (World Health Organization and Columbia University, 2016). Supervisors rated each session component as ‘superior’, ‘satisfactory’, ‘needs improvement’, ‘failed to attempt’, ‘not applicable’, or ‘could not assess’. Fidelity was calculated as the percentage of components rated superior or satisfactory. We used the follow-up rate and percentage of missing data to assess the feasibility of study procedures.

### Outcome measurements

We assessed adolescent mental health outcomes at three time points: immediately before the first individual session (baseline); within 2 weeks of the 12th group session (post-treatment); and 8–10 weeks after the 12th group session (follow-up). Primary outcomes were depression measured with the DSRS, and functional impairment measured with a locally developed tool. The DSRS is an 18-item screening tool for children and adolescents that has been used in various cultural contexts (Ivarsson *et al*., 1994; Denda *et al*., 2006; Panter-Brick *et al*., 2009). In the adapted Nepali version items are presented as questions, for example ‘Are you able to sleep well?’, ‘Do you feel like eating when you see food?’ and response options are ‘never’, ‘sometimes’, or ‘mostly’ (Kohrt *et al*., 2011). Responses were summed to give a total score out of 36 with higher scores indicating more severe depression. The functional impairment tool is a 10-item scale to assess adolescents' ability to participate in the past 2 weeks in locally relevant daily activities including working in the fields, doing house chores, and spending time with friends (Jordans *et al*., 2010). Items are in question format and responses are ‘none of the time’, ‘a little of the time’, ‘some of the time’, and ‘all the time’. Scores range from 0 to 30 with higher scores indicating more impairment. The tool was originally developed for a study in southwestern Nepal using methods outlined by Bolton and Tang (2002). We adapted it for Sindhupalchowk using free listing to identify tasks that are important to adolescents in this setting. Secondary outcomes were anxiety, PTSD, and disruptive behaviour, measured with the Beck Anxiety Inventory (BAI, 21 items); Child PTSD Symptom Scale (CPSS) (17 items); and Disruptive Behaviour International Scale – Nepal (DBIS-N, 24 items) (Kohrt *et al*., 2003; Jordans *et al*., 2010; Burkey *et al*., 2016; Burkey *et al*., 2018). Tools were translated into Nepali and previously validated in Nepal.

We wanted to use different tools to assess the primary outcome and in-session depression assessments because we anticipated adolescents would become very familiar with the latter, increasing the risk of social desirability bias and a testing effect. We also wanted a shorter tool in the sessions to mitigate interview fatigue. We therefore used the Patient Health Questionnaire – Adolescent Version (PHQ-A, 9 items) to monitor session-by-session change (Johnson *et al*., 2002). The PHQ-A is a modification of the Patient Health Questionnaire which was validated in Nepal among adults (Kohrt *et al*., 2016). Validation of the PHQ-A among adolescents in Nepal is underway.

### Cost analysis

We did an activity-based cost analysis of the design and implementation of IPT from the provider perspective. Costs from monthly project accounts were entered in an Excel tool, divided into start-up or implementation costs, and allocated to different cost centres (capital, staff, and materials) and intervention activities (e.g. adaptation, training, facilitation, etc.). Interviews with project staff before implementation and at follow-up informed how they divided their time across activities and thus how to allocate their salaries. The total cost of the intervention was annualised then divided by the number of participants in a year to estimate a unit cost per IPT participant. We calculated a unit cost per participant based on four assumptions. First, the IPT manual, training materials, and training of trainers were treated as single capital items with a useful life of 7 years (Greco *et al*., 2018). Like other capital costs, these were annualised over their useful life using a discount rate of 8.00% (Nepal Government Bond yield in 2020) (Greco *et al*., 2018; International Monetary Fund, 2020). Second, each facilitator pair can run three groups of 10 adolescents simultaneously, and a total of 36 groups per year (360 adolescents). Third, facilitators need annual refresher training and supervisors need sporadic support from master trainers. Fourth, when estimating unit costs for 360 participants, we assumed a linear increase of the recurrent costs incurred to provide the intervention to 62 participants. This is because sessions would run in the same place and additional participants would come from the same area with similar accessibility. All costs were inflated to 2020 and converted from Nepali rupees to international and US dollars using World Bank data on consumer price index and exchange rates (World Bank, 2020). Last, univariate sensitivity analyses were carried out to test the key assumption and cost driver relating to the annual number of participants that facilitators could serve at capacity.

### Quantitative data collection and analysis

We based the sample size on the minimum number of groups needed to pilot various facilitator combinations (e.g. both female facilitators, both male, one female, and one male). Facilitators recorded attendance and adolescents completed the PHQ-A at each session. We collected quantitative data on mental health, costs, and sociodemographic characteristics through interviews with adolescents at baseline, post-treatment, and follow-up. Researchers conducted the interviews using tablets programmed with ODK data collection software.

Mental health outcomes were analysed as continuous outcomes. We conducted descriptive analyses comparing outcomes at baseline to post-treatment, and baseline to follow-up and calculated a measure of effect size (Cohen's *d*). We repeated these analyses excluding three participants referred to the psychosocial counsellor to see how it affected outcomes. We conducted sub-group analyses to see if changes in outcomes were concentrated by gender, caste/ethnic group, income level (low *v.* not low, with low defined as sufficient family income for no more than 6 months), family type (nuclear or extended), baseline mental health comorbidities, IPT group, and school.

### Qualitative evaluation of feasibility

Researchers conducted semi-structured interviews with 16 adolescents and all six facilitators. We sampled female and male adolescents with low (less than four group sessions), medium (4–7), and high attendance (8–12) at group sessions, across age and caste/ethnic groups. Topic guides included questions on positive and negative aspects of IPT and adverse events. Interviews were in Nepali, audio-recorded, transcribed, and translated into English. Data were analysed using the Framework approach which involved developing a coding framework used to open-code interview transcripts, and mapping summaries into a matrix (Excel spreadsheet) (Gale *et al*., 2013).

### Impact of COVID-19 on implementation and data collection

Face-to-face research in Nepal was banned in March 2020 so we had to conduct 25/62 post-treatment surveys and all follow-up surveys by phone. Using regression analysis with interactions, we tested whether the change in mental health outcomes between post-treatment and follow-up differed by whether the post-treatment interview was conducted on the phone or in person. We found no statistically significant differences in any outcome between individuals who had their post-treatment interview conducted in person *v.* over the phone.

Supervisors were unable to complete all planned observations of sessions and supervisions were done by phone. We conducted qualitative interviews with facilitators using phone/video conferencing. Qualitative interviews with adolescents were delayed until after the lockdown in June 2020. Due to COVID-related restrictions some IPT groups did not receive 12 in-person group sessions: one male group received 11 group sessions; one male group received 9/12 in-person group sessions with the final session conducted individually by phone; and one male group received 10 in-person group sessions plus a final session by phone.

### Ethics

The Nepal Health Research Council (637/2018) and King's College London Research Ethics Committee (RESCM-18/19–8427) approved the research.

## Results

Table 1 presents adolescent baseline characteristics. More than two-thirds were from low-income households defined as those having sufficient income for no more than 6 months. Mean scores for depression, anxiety, PTSD, and disruptive behaviour were similar across genders. Baseline characteristics by school are reported as online Supplementary materials (Appendix A). The intra-class correlation coefficient for DSRS at baseline across schools was 0.07.

**Table 1.** Baseline descriptive sample characteristics, overall and by gender

	Total (*n* = 62)	Girls (*n* = 30)	Boys (*n* = 32)
Mean outcome scores (s.d.)			
Depression self-rating	17.21	18.23	16.25
	(2.96)	(3.42)	(2.1)
Functional impairment	12.55	12.57	12.53
	(4.5)	(4.71)	(4.37)
Patient Health Questionnaire	10.00	10.67	9.38
	(3.96)	(4.17)	(3.71)
Beck Anxiety Inventory	16.68	18.63	14.84
	(7.11)	(7.84)	(5.89)
Child PTSD	25.03	27.23	22.97
	(7.51)	(7.82)	(6.67)
Behavioural index	6.19	6.4	6
	(3.48)	(3.83)	(3.17)
Covariates (% in each group)			
Age (mean, s.d.)	14.9 (1.2)	15.3 (1.3)	14.6 (1.0)
Caste: Brahman/Chhetri	41.9 (*n* = 26)	40.0 (*n* = 12)	43.8 (*n* = 14)
Janajati	53.2 (*n* = 33)	53.3 (*n* = 16)	53.1 (*n* = 17)
Dalit	4.8 (*n* = 3)	6.7 (*n* = 2)	3.1 (*n* = 1)
Low income^a^ (*v.* not low)	69.4 (*n* = 43)	73.3 (*n* = 22)	65.6 (*n* = 21)
Nuclear family (*v.* extended)	59.7 (*n* = 37)	60.0 (*n* = 18)	59.4 (*n* = 19)
Living with parents (*v.* no)	66.1 (*n* = 41)	60.0 (*n* = 18)	71.9 (*n* = 23)

s.d., standard deviation.

a Low income = family income sufficient for no more than 6 months.

### Feasibility of IPT and study procedures

The participation rate was 93.9% (62/66) and mean attendance was 82.3% [standard deviation (s.d.) 18.9, *n* = 62]. Online Supplementary Appendix B is a participant flowchart. Supervisors rated 95.3% of IPT-specific tasks as superior or satisfactory across 27/90 sessions observed. All adolescents were followed up at post-treatment and follow-up. There were no missing data.

### Outcome trends

**Fig. 1.**
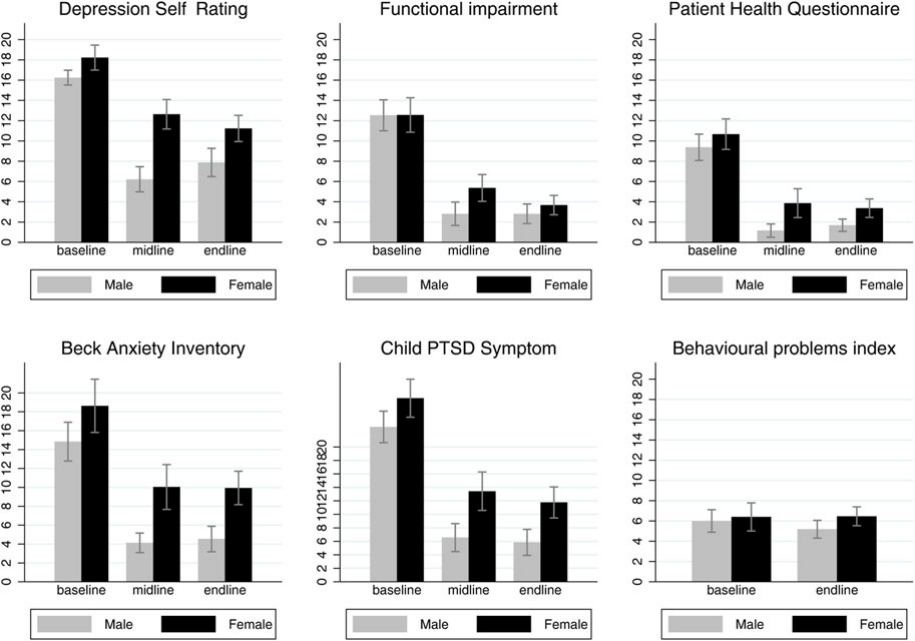
Outcome means and 95% confidence intervals by gender at baseline, post-treatment, and follow-up time points.

Table 2 and Fig. 1 show that all outcomes except for disruptive behaviour improved between baseline and post-treatment, and between baseline and follow-up. Facilitators referred three adolescents at high risk of suicide to a psychosocial counsellor. Results remained the same after excluding these participants from the analyses. Most improvements happened between baseline and post-treatment. Cohen's *d* ranged from 1.5 for anxiety to 2.5 for functional impairment, indicating large effect sizes. Across outcomes, fidelity to the IPT manual and effect size were not correlated.

**Table 2.** Results overall, and by gender with within-group effect sizes (Cohen's *d*)

	Baseline (1)	Post-treatment (2)	*d* size (2) − (1)	Follow-up (3)	*d* size (3) − (1)
Depression self-rating scale	17.21 (16.46; 17.96)	9.32 (8.06; 10.58)	1.9	9.50 (8.45; 10.55)	2.1
Male	16.25 (15.49; 17.01)	6.21 (4.93; 7.50)	3.4	7.85 (6.44; 9.31)	2.6
Female	18.23 (16.96; 19.51)	12.63 (11.13; 14.13)	1.5	11.23 (9.89; 12.58)	1.9
Functional impairment	12.55 (11.40; 13.69)	4.05 (3.11; 4.99)	2.1	3.23 (2.53; 3.92)	2.5
Male	12.53 (10.95; 14.11)	2.81 (1.62; 4.00)	2.5	2.81 (1.81; 3.81)	2.7
Female	12.56 (10.81; 14.33)	5.37 (4.00; 6.74)	1.7	3.67 (2.67; 4.66)	2.3
Patient Health Questionnaire	10.00 (8.99; 11.01)	2.47 (1.62; 3.31)	2.1	2.50 (1.92; 3.08)	2.3
Male	9.37 (8.04; 10.71)	1.16 (0.48; 1.84)	2.8	1.69 (1.06; 2.31)	2.7
Female	10.67 (9.11; 12.23)	3.87 (2.39; 5.34)	1.7	3.36 (2.42; 4.31)	2.1
Beck Anxiety Inventory	16.68 (14.87; 18.48)	6.98 (5.50; 8.46)	1.5	7.15 (5.84; 8.45)	1.5
Male	14.84 (12.72; 16.97)	4.13 (3.05; 5.20)	2.3	4.53 (3.13; 5.93)	2.1
Female	18.63 (15.70; 21.56)	10.03 (7.57; 12.49)	1.2	9.93 (8.09; 11.77)	1.3
Child PTSD Symptom Scale	25.03 (23.12; 26.94)	9.89 (7.92; 11.85)	2.0	8.71 (7.03; 10.39)	2.3
Male	22.97 (20.56; 25.38)	6.56 (4.42; 8.71)	2.6	5.84 (3.85; 7.84)	2.8
Female	27.23 (24.31; 30.16)	13.43 (10.48; 16.39)	1.8	11.77 (9.38; 14.15)	2.2
Disruptive Behaviour International Scale Nepal	6.20 (5.30; 7.07)			5.80 (5.14; 6.46)	0.1
Male	6.00 (4.86; 7.14)			5.19 (4.28; 6.09)	0.3
Female	6.40 (4.97; 7.83)			6.47 (5.51; 7.42)	0.0

**Fig. 2.**
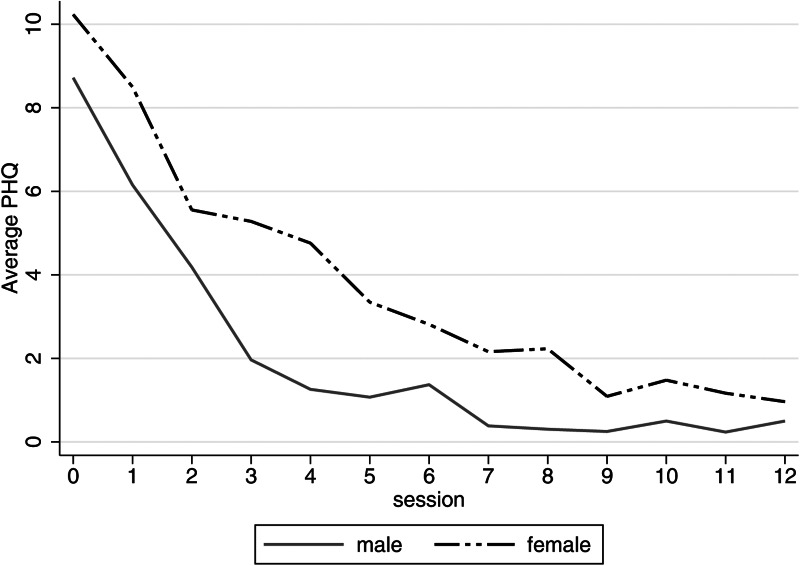
Session by session depressive symptoms by gender.

The rate of improvement was highest in the first three sessions and boys improved faster than girls (Fig. 2). Males and females had similar levels of depression, anxiety, and PTSD symptoms at baseline, both groups improved up to follow-up, but males improved more than females across these outcomes (Table 2 and Fig. 1).

Improvements in mental health did not differ by caste/ethnic group, income level, or family type. Graphs of outcomes by age (13–14 *v.* 15–19 years) and number of comorbidities are given in online Supplementary Appendices C and D. Adolescents in schools 3 and 4 improved more than those in schools 1 and 2 for all outcomes except for disruptive behaviour. Because schools 3 and 4 ran male-only groups, it was unclear if differences in mental health reflected differences in improvement due to gender or the school environment. All eight IPT groups improved from baseline to follow-up across depression, anxiety, and PTSD outcomes with some exceptions: depression measured using the DSRS did not improve in one female group, and anxiety did not improve in two female groups.

### Cost analysis

The total intervention cost was NPR 6 682 849 (Int'l $199 310; US $57 457) between September 2018 and 2020, with an annualised cost of NPR 3 341 424 (Int'l $99 655; US $27 728; online Supplementary Appendix E). The unit cost was NPR 53 894 (Int'l $1607; US $447.2) per participant (*n* = 62). If facilitators were operating at capacity (*n* = 360) the annualised total cost is NPR 4 055 368 (Int'l $120 947; US $34 867) and unit cost is NPR 11 265 (Int'l $336.0; US $96.9). The main driver of unit cost is the number of groups a pair of facilitators can run at capacity. Assuming they run two groups simultaneously (*n* = 240) the unit cost is NPR 16 470 (Int'l $491.2; US $141.6); if they run four groups (*n* = 480) the unit cost is NPR 8663 (Int'l $258.4; US $74.5).

### Perceptions of acceptability, feasibility, and utility

Qualitative interviews captured adolescents' mixed feelings about being recruited to IPT (Table 3). Nine felt happy to join. One girl initially worried that the programme was a front for human trafficking. Others, especially girls, were worried they would damage their ‘family prestige’ (*pariwarko Ijjat janu*) by sharing about their family in a group. However, having completed the programme all adolescents emphasised the value of sharing and the advice and support they received from group members.

**Table 3.** Findings from qualitative interview with adolescents and facilitators

Theme	Category	Quote
Utility		
	Problems helped by IPT	‘People who fight, quarrel, have heart mind related problems due to the poor relationship with parents, also those who have poor relationships with their friends, etc. would benefit the most’. Male 14 ‘I used to fight with my friends […]. In that program they taught us about the proper ways of talking and explaining to friends at such times. It helped me a lot’. Male 14
	Impact of IPT on adolescents' lives	‘The sessions helped us; it helped us with the fights at home. My friends [in the group] used to tell me that they no longer feel alone’. Female 16 ‘It had a positive effect on everything. My studies got better along with the relationship with my friends. The relationship with my family also got better’. Male 15
	Usefulness and suitability of IPT technique	‘I liked IPT very much. As we started the training, I felt it was new and I got excited and curious to know more about it. I wasn't confident in the beginning to conduct the session, but later as we proceeded, their problems lessened. They took our suggestions positively. These things make me feel very happy. I have realised that IPT is a very good technique, I had also used some of the techniques to relieve stress. It's not just limited to managing depression, but also helps to live life better for those who know the techniques. We get to learn ways to manage anger and stress that we have with other people’. Facilitator ‘For instance, let's take “dispute/argument” as a problem. There we were taught to implement interpersonal skill development. We have to implement that technique when people have problems in relationships. The anger management technique was very effective. Clients had also implemented it in their real lives as most of them had this problem. It was the most implemented technique. Thus, techniques such as anger management and interpersonal skill development were very effective for participants’. Facilitator ‘Every solution comes by discussing with friends. We can tell them if we have any solution to their problems, similarly they can also do the same. If it doesn't help entirely, it would surely help in some way. That way, they would feel good as well’. Male 14
Acceptability		
	Feelings about joining IPT	‘At first, when I was unaware, I thought that [facilitators] had come from outside [the community] and might sell us. Later, I participated in the weekly meetings and began sharing about heart mind problems […]. I was happy sharing with them’. Female 16
	Positive and negative feelings about participating in IPT	‘All of us could share about heart mind related problems in the group, we could also make fun of each other, laugh, and play games as well. […] If someone gets sad then everyone can create a cheerful environment and that person can speak cheerfully. That's why I could be happy with the help from my friends. We used to play as well’. Male 15 ‘It is beneficial in a group. If we had to sit alone then we would have been unaware about other peoples’ problems. If I were alone in the session and if I were the only one with such problems, then I would have been confused about what needs to be shared. But since everyone would share in the group, we could feel normal knowing that everyone was suffering with the same problem’. Female 16 ‘It was nice. I could share my heart mind related problems with my friends and listen to theirs’ as well. My friends in the group suggested various ways to solve the problems and I could also suggest them’. Male 15 ‘Everyone listened to each other's problems. If we hadn't participated in the session, then everything would have been within ourselves and stuck within us. That would have made us very sad and unhappy. In those sessions, we could listen to each other's problems and realise that it's not just me but others as well with such similar problems. […] I felt happy knowing all those things’. Female 16
	Identifying reasons for absences	‘Going [to the sessions] I could relieve my heart mind problems. That's the reason that made me feel like going. Also, we used to have fun during the sessions’. Female 16 ‘The reason for their absence was that they had household work, some were sick, some did not come to school, while some had informed us that they couldn't join the session as they had some emergency work at home’. Facilitator ‘We […] asked the participant's reason for not joining the session. They said that their problems were minor compared to others and they didn't like participating in the session. They also lived far away, so it was difficult as they had to get assistance to get home. So, having to go alone, covering such a long distance, that person left the session’. Facilitator
	Acceptability of facilitators	‘They are very close to us. The facilitators are like my sisters. It was very comfortable being in the session with them’. Female 16 ‘I liked [the facilitators] very much. They understood us, they weren't like others who get lost and ignore us whilst we are speaking or divert the conversation in between. They weren't like our teachers but friends. They were fun and they made it easy to express everything. They listened to us and provided suggestions. That's why I liked them a lot’. Female 16
Fidelity		
	Facilitators' preparedness for sessions	‘I was prepared as I had taken 20 days of training before. All of us had difficulties and it's natural to have trouble in the beginning. As we kept on working, we gradually gained the experience from the practice group, which trained us for the real group session. […] We had to look up in the manual to prepare ourselves. We sensed that we must be prepared a lot, such feelings also made us prepared. We had two days [refresher] training before the real session. At that time [our supervisors] had said that we did well’. Facilitator
	Deviations from the IPT manual	‘Sometimes, when we talk about things, we might need to go outside the content in the manual. For instance, there are no techniques for those who take marijuana, weed, etc. and we have to go beyond the manual to deal with such cases’. Facilitator
Implementation		
	Suitability of school as the venue	‘My friends used to peep inside the room, but later as we began locking the door, they stopped disturbing us. Also, our sessions used to take place during school hours, so it went smoothly’. Male 14 ‘There was one sister in the village who had left school. I wanted to bring her to the session and so I was planning to tell [the facilitator] the next time she came to the village, but unfortunately it was too late, and she committed suicide. She had some problems at home. I feel very bad about that. If only I could have taken her to the session, I would have saved her life. […] I think you shouldn't just ask about the cases [of depression] in school but ask in the village as well’. Female 16
	Suitability of group size	‘The number [of people in the group] is good: eight people is enough. If there were more it would be disturbing. With eight it will be fun to share. If there are more people, then some may not share, some may be shy. There were some shy people in my group but when the facilitators assured them a safe environment for sharing […] they began sharing in the group’. Female 16 ‘There were just four clients in the beginning but now there are many. Due to this, there wasn't enough time to talk about everyone's problems. We used to ask if anybody else had similar problems and gave priority to those who had problems. For the following week we used to talk to people based on their PHQ score’. Facilitator
	Ideal timing of sessions	‘For me, the morning hours are best… You may also manage the evening hours, after school but those who live far away will have to walk a long distance and return alone. All their friends would have gone by that time, and it would be difficult on the way home’. Female 16 ‘My study was affected a bit as I had to go to the session during the classes. It happened two or three times’. Female 15
Feasibility		
	Perceptions of programme duration	‘I told them [about my problem] in the eighth or ninth session but didn't share before that. I gave them a hint but didn't share everything. I didn't have the courage to share. I don't share that easily. This was my first time sharing with someone’. Female 16 ‘Our heart mind problems would alleviate in 12 weeks, however for some people eight sessions would be enough’. Male 15 ‘I think that 12 sessions are needed for us. I think it's fine to have 12 sessions…I need them, so do others. We may not have shared about all our problems, and we might have kept things within us. In the beginning, it becomes difficult sharing, as we get shy and scared to share. I think that we can share about our problems in 12 sessions’. Female 16
	Experiences of involving parents in the programme	‘The advantages that I saw were that the adolescents didn't always have cell phones with them, so when we had to call them for the session, we would rather call their parents, and they would inform their kids about it. The participants feel that if us facilitators will call their parents then they would easily let their kids go. Even if the participants reached home late after the session or left early in the morning for the session, their parent wouldn't scold them. That's why I think that family meetings are important’. Facilitator ‘There wasn't any problem as such because we had already visited adolescents even before we met the parents. During the second meeting the clients would be together with parents, which might be a problem for the clients, as they would be scared all the time that we facilitators might say something about their heart-mind problems in front of their parents. But we had already mentioned to them about confidentiality, promising them that we will not mention anything if it includes the problem with the parents. That's why there wouldn't be any problem as such’. Facilitator
Adverse events		
	Experiences of adverse events	‘There were one or two people from among seven or eight who had suicidal tendencies, and they were from both genders. [We] felt that it was more prevalent among females than males. We used to work according to the [IPT] manual and carry out the processes but didn't find any serious cases among the male participants. The clients used to share with us about the reasons for getting such thoughts. They had attempted suicide once but didn't do it again. And when they were asked if they currently had such thoughts they replied saying that they didn't. Also, when asked about the reasons for wanting to live they had some answer for that, which made them realise that they shouldn't attempt such action again’. Facilitator ‘There was one client who had suicidal thoughts, and I talked a lot with that person. At that time, I couldn't focus, felt heavy and became tired… I didn't feel good. But as I started hanging out with friends, I got better and felt happy. […] I used to be scared that if someone with such tendencies might commit suicide. So, I could take it easy only after making them fill the “Safety Form” and talking with them’. Facilitator
Contagion		
	Dissemination of IPT techniques beyond participants	‘I didn't share my heart mind problems with my friends in the past but now they call me on my phone and share their feelings. They had told me that the program helped them as well’. Female 16 ‘I used to tell them that it's related to heart and mind, for instance the problem arises when someone quarrels at home and is dominated by other people. I explained to them that way’. Female 15

Adolescents thought IPT sessions were ‘fun’ and enjoyed the singing and games. They were unanimously positive about facilitators whom they described as like ‘friends’ or siblings who understood their problems, listened, and created a comfortable group environment. Adolescents and facilitators thought the most useful skills were in decision-making, communicating, role-play, anger management, and relaxation. Facilitators described how they competently managed adolescents with suicidal ideation, but how it made them feel ‘heavy’ and anxious. Adolescents said that even though the programme had finished, they continued to use and share the skills they learned to help themselves and their friends.

School was perceived to be a suitable and convenient location for IPT sessions though some adolescents reported being disturbed by noise and concerns about privacy. They suggested sessions should be in a room with a lockable door and scheduled for the early morning or lunchtime to avoid having to miss class or return home late. A female participant highlighted the need to include out of school adolescents in groups:

‘There was one sister in the village who had left school. I wanted to bring her to the session and so I was planning to tell [the facilitator] but unfortunately it was too late and she committed suicide. I feel very bad about that. If only I could have taken her to the session I would have saved her life. […] I think you shouldn't just ask about the cases [of depression] in school but ask in the village as well’. Female 16

Adolescents and facilitators agreed that IPT participants with more *heart-mind* problems required more sessions than those with fewer problems. All facilitators and some adolescents recommended reducing the number of sessions. Facilitators thought most participants' problems were resolved after session eight and that they were less likely to attend subsequent sessions. Facilitators identified illness, exams, school/home/paid work, festivals, and sporting events as reasons for absence.

Most parents supported their child's participation and reminded them to attend. Facilitators stressed the importance of initially meeting parents to build rapport and learn more about the adolescent but found it difficult to travel to remote villages and find a mutually convenient time.

Fourteen adolescents said they had not experienced stigma related to participating in IPT. One said that friends had teased him, and another described how a teacher shouted at him for missing class. In one school facilitators asked the principal to intervene because a teacher referred to a participant as ‘psycho’ and IPT groups as ‘psycho groups’. Adolescents and facilitators recommended school-level psychoeducation and programme orientation to explain that IPT can help adolescents solve problems and improve their studies.

## Discussion

The results of the study support progression to an RCT to evaluate the effectiveness and cost-effectiveness of IPT for adolescents with depression in Nepal. Adolescents found IPT acceptable, evidenced by high recruitment and attendance rates and qualitative data suggesting they thought groups were useful and fun. Adolescents perceived that IPT reduced their heart-mind problems and outcome trends suggest improvements in depression, functioning, anxiety, and PTSD symptoms between baseline and follow-up. Training and supervision of nurses and lay workers to deliver IPT was feasible, evidenced by high levels of fidelity to the manual. The recruitment process identified sufficient adolescents with depression, although recruitment of more out of school adolescents is needed. Ethics and safety procedures ensured high risk suicidal adolescents were identified and treated. Data collection procedures were feasible evidenced by no missing data. A cluster RCT which randomises schools rather than individuals to trial arms could help to minimise any contamination related to adolescents sharing IPT skills with their peers.

There are several strengths of our study including the mixed methods approach used to test a culturally adapted psychological treatment that is potentially scalable through the education system. The lack of a control arm means we cannot infer causality and attribute adolescents' improvements in mental health to IPT. Although outcome assessments were conducted by research assistants, not IPT facilitators, we cannot exclude the possibility of social desirability bias in surveys and qualitative interviews. COVID-19 lockdown measures in 2020 halted face-to-face IPT sessions and data collection but it was still possible to conduct sessions and assessments with adolescents by phone. Moreover, despite the unprecedented and potentially distressing circumstances during lockdown, we observed maintained improvements in participants' mental health.

Our study is relevant beyond Nepal and contributes to a growing body of global research showing the feasibility of psychological treatments delivered by people who are not specialists in mental health (Clarke *et al*., 2013; Cuijpers *et al*., 2018; van Ginneken *et al*., 2021). Nurses and lay workers delivered IPT in pairs. This facilitator model is advantageous because the intervention could be scaled up through the education system and network of school-nurses. Recruitment of male lay workers to support the mainly female workforce of school nurses ensures the intervention is acceptable to adolescents who prefer a facilitator of the same gender. We did not directly compare therapeutic competency between nurses and lay workers because of the small number of facilitators in the study. However, this analysis could help to optimise facilitator training and supervision and should be part of a future fully powered RCT.

We delivered IPT through secondary schools. Adults and adolescents who participated in our formative work said they did not trust organisations from outside the community so we should deliver the intervention in schools (Rose-Clarke *et al*., 2020). The high rate of attendance at group sessions suggests school is a convenient and accessible location for adolescents. This contrasts with trials of IPT in healthcare settings reporting low uptake (51%) and high dropout rates (28%) in South Africa and Kenya, respectively (Petersen *et al*., 2014; Meffert *et al*., 2021). Evidence for school-based mental health interventions in LMICs is only just emerging but results are promising (Fazel *et al*., 2014; Michelson *et al*., 2020; Bradshaw *et al*., 2021). Schools vary greatly in terms of environment, resources, and their suitability as a venue for mental health care. In Nepal, corporal punishment is common, and schools may not have a quiet, private room for group sessions. Adolescents participating in a mental health intervention may experience stigma and discrimination from peers and teachers (Gronholm *et al*., 2018). Participant confidentiality and safety are paramount so assessment of a school's suitability and readiness, and parallel anti-stigma and psychoeducation activities must be pre-requisites for school-based psychological intervention in Nepal and beyond.

For all the potential advantages of school-based programmes (accessibility, scalability, and efficacy), they usually exclude adolescents who are not in education. In Nepal this is around 12% of adolescents who tend to be from poorer households, are at greater risk of early marriage and child labour and are likely to have more mental health problems than school-going adolescents (Burgess *et al*., 2022). Although we did not explicitly exclude out of school adolescents, we did not proactively search for them. Earlier formative research suggests that out of school adolescents would consider attending school-based programmes alongside school-going adolescents. One possible solution could therefore be a more inclusive community-based recruitment strategy. This could involve training adolescents, community leaders, and health workers to use the Community Informant Detection Tool to find and refer out-of-school adolescents (Jordans *et al*., 2015). Elsewhere, IPT sessions have been held in community healthcare settings which could be feasible in parallel or combined with sessions in schools (Patel *et al*., 2010; Petersen *et al*., 2012).

The main aim of our study was to assess the feasibility and acceptability of IPT, rather than its effects. We report large effect sizes for mental health outcomes but these are likely inflated due to the pre-post study design without a control group and clustering at the school level which, with only four schools, we could not account for in the analysis (Bryan and Jenkins, 2015). More modest effects have been reported in RCTs of adolescent IPT (Mufson *et al*., 1999; Mufson *et al*., 2004*b*; Bolton *et al*., 2007; Tang *et al*., 2009). Our strategy of selecting the six most competent facilitators to deliver the intervention from the nine we trained likely facilitated the study outcomes and may not be feasible in a larger community-based programme. However, there are several reasons to suggest IPT may be effective in rural Nepal. First, IPT was the result of an evidence-based cultural adaptation of IPT in this setting (Rose-Clarke *et al*., 2020). Evidence from meta-analyses suggests therapies that have been culturally adapted may be more effective than those that have not (Benish *et al*., 2011; Hall *et al*., 2016). Second, the group-based delivery of IPT facilitated locally relevant reciprocal support, empathy, and compassion, which participants valued highly (Kohrt *et al*., 2020). Third, experiences of physical and sexual abuse are prevalent among adolescents in Nepal [Ministry of Health and Population (MoHP), 2012]. Research suggests IPT may be particularly helpful for those with a history of maltreatment and/or trauma (Betancourt *et al*., 2012; Toth *et al*., 2020). Last, Gunlicks-Stoessel *et al*. (2010) found greater benefits of IPT among adolescents reporting high levels of conflict with their mother or peers (Gunlicks-Stoessel *et al*., 2010). Our qualitative data suggest many IPT participants experienced such difficulties and found communication analysis and anger management techniques particularly useful (Rose-Clarke *et al*., 2021). Whether the lack of correlation between fidelity to IPT and mental health outcomes is due to a ceiling effect or because adolescents mainly benefitted from the supportive group environment rather than the IPT content should be explored through an RCT.

We observed a greater improvement in depression among male than female participants which warrants further investigation in an RCT powered to detect gender differences. We aimed to recruit an equal number of male and female participants, but it was harder to find males with a sufficiently high DSRS score. This is likely because the prevalence of depression is lower among males, with the gender difference peaking in adolescence (odds ratio 3.02 between the ages of 13 and 15) (Hyde and Mezulis, 2020). Whether this gender difference is due to boys under-reporting depression because it is perceived to be unmasculine, or because distress in boys manifests more in externalising symptoms that are not captured by the DSRS remains unclear (Chaplin and Aldao, 2013; Hyde and Mezulis, 2020; Rice *et al*., 2021).

Qualitative data suggest some adolescents and facilitators preferred fewer than 12 group sessions. This is supported by data showing that the rate of reduction in depressive symptoms was highest in the first three sessions and symptoms stabilised around the ninth group session. IPT was originally designed to be delivered over 12–16 sessions although it has been delivered in eight group sessions (Markowitz and Weissman, 2004; Swartz *et al*., 2014; World Health Organization and Columbia University, 2016). Alternative intervention models with fewer sessions and/or additional sessions for adolescents with persistent symptoms could be more acceptable and cost-effective. However, reducing the number of sessions reduces time for participants to consolidate IPT skills and techniques, which could reduce effect sizes and any longer-term treatment benefits. Moreover, in a two-stage model, adolescents requiring additional sessions may feel a sense of failure and lose hope of recovery. Research is therefore needed to explore IPT treatment duration as a potential effect modifier.

Taken at face value, unit costs for IPT in Nepal appear high, but our estimates are in line with those reported in a cluster-randomised trial of a group psychotherapy intervention for adults living with HIV with mild to moderate depression in rural Uganda (Nakimuli-Mpungu *et al*., 2020). This intervention reduced depression and was highly cost-effective: the study estimated that costs would have to increase by 1000% before the intervention stopped being cost-effective. While it is challenging to predict costs at scale, this evidence suggests that IPT in Nepal could be cost-effective. Moreover, additional adjustments such as reducing the number of group sessions would reduce costs further.

## Conclusion

A culturally adapted version of group IPT for adolescents delivered through schools in Nepal is feasible and acceptable. Our results support progression to an RCT to assess the efficacy and cost-effectiveness of IPT in this setting. Research is needed to develop alternative recruitment strategies for out of school adolescents, and to explore how treatment duration and social factors could modify IPT's effects to optimise intervention delivery and impact.

## Supplementary material

For supplementary material accompanying this paper visit https://doi.org/10.1017/gmh.2022.46. click here to view supplementary material

## References

[ref1] Barbui C, Purgato M, Abdulmalik J, Acarturk C, Eaton J, Gastaldon C, Gureje O, Hanlon C, Jordans M, Lund C, Nosè M, Ostuzzi G, Papola D, Tedeschi F, Tol W, Turrini G, Patel V and Thornicroft G (2020) Efficacy of psychosocial interventions for mental health outcomes in low-income and middle-income countries: an umbrella review. The Lancet. Psychiatry 7, 162–172. 31948935 10.1016/S2215-0366(19)30511-5

[ref2] Benish SG, Quintana S and Wampold BE (2011) Culturally adapted psychotherapy and the legitimacy of myth: a direct-comparison meta-analysis. Journal of Counseling Psychology 58, 279–289. 21604860 10.1037/a0023626

[ref3] Betancourt TS, Newnham EA, Brennan RT, Verdeli H, Borisova I, Neugebauer R, Bass J and Bolton P (2012) Moderators of treatment effectiveness for war-affected youth with depression in northern Uganda. The Journal of Adolescent Health 51, 544–550. 23174463 10.1016/j.jadohealth.2012.02.010

[ref4] Bolton P and Tang AM (2002) An alternative approach to cross-cultural function assessment. Social Psychiatry and Psychiatric Epidemiology 37, 537–543. 12395144 10.1007/s00127-002-0580-5

[ref5] Bolton P, Bass J, Betancourt T, Speelman L, Onyango G, Clougherty KF, Neugebauer R, Murray L and Verdeli H (2007) Interventions for depression symptoms among adolescent survivors of war and displacement in northern Uganda: a randomized controlled trial. JAMA 298, 519–527. 17666672 10.1001/jama.298.5.519

[ref6] Bradshaw M, Gericke H, Coetzee BJ, Stallard P, Human S and Loades M (2021) Universal school-based mental health programmes in low- and middle-income countries: a systematic review and narrative synthesis. Preventive Medicine 143, 106317. 33159922 10.1016/j.ypmed.2020.106317

[ref7] Bryan ML and Jenkins SP (2015) Multilevel modelling of country effects: a cautionary tale. European Sociological Review 32, 3–22.

[ref8] Burgess RA, Jeffery M, Odero SA, Rose-Clarke K and Devakumar D (2022) Overlooked and unaddressed: a narrative review of mental health consequences of child marriages. PLoS Global Public Health 2, e0000131. 10.1371/journal.pgph.0000131 PMC10021205 36962120

[ref9] Burkey MD, Ghimire L, Adhikari RP, Kohrt BA, Jordans MJ, Haroz E and Wissow L (2016) Development process of an assessment tool for disruptive behavior problems in cross-cultural settings: the Disruptive Behavior International Scale – Nepal version (DBIS-N). International Journal of Culture and Mental Health 9, 387–398. 28093575 10.1080/17542863.2016.1226372 PMC5234690

[ref10] Burkey MD, Adhikari RP, Ghimire L, Kohrt BA, Wissow LS, Luitel NP, Haroz EE and Jordans MJD (2018) Validation of a cross-cultural instrument for child behavior problems: the disruptive Behavior International Scale – Nepal version. BMC Psychology 6, 51–51. 30390713 10.1186/s40359-018-0262-z PMC6215604

[ref11] Chaplin TM and Aldao A (2013) Gender differences in emotion expression in children: a meta-analytic review. Psychological Bulletin 139, 735–765. 23231534 10.1037/a0030737 PMC3597769

[ref12] Clarke K, King M and Prost A (2013) Psychosocial interventions for perinatal common mental disorders delivered by non-mental health specialists in low and middle-income countries: a systematic review and meta-analysis. PLoS Medicine 10, e100154. 10.1371/journal.pmed.1001541 PMC3812075 24204215

[ref13] Clarke A, Sorgenfrei M, Mulcahy J, Davie P, Friedrich C and Mcbride T (2021) Adolescent mental health: a systematic review on the effectiveness of school-based interventions. Early Intervention Foundation Available at https://www.eif.org.uk/report/adolescent-mental-health-a-systematic-review-on-the-effectiveness-of-school-based-interventions (Accessed 16th August 2022).

[ref14] Cuijpers P, Karyotaki E, Reijnders M, Purgato M and Barbui C (2018) Psychotherapies for depression in low- and middle-income countries: a meta-analysis. World Psychiatry 17, 90–101. 29352530 10.1002/wps.20493 PMC5775122

[ref15] Cuijpers P, Karyotaki E, Eckshtain D, Ng MY, Corteselli KA, Noma H, Quero S and Weisz JR (2020) Psychotherapy for depression across different age groups: a systematic review and meta-analysis. JAMA Psychiatry 77, 694–702. 32186668 10.1001/jamapsychiatry.2020.0164 PMC7081149

[ref16] Denda K, Kako Y, Kitagawa N and Koyama T (2006) Assessment of depressive symptoms in Japanese school children and adolescents using the Birleson Depression Self-Rating Scale. International Journal of Psychiatry in Medicine 36, 231–241. 17154151 10.2190/3YCX-H0MT-49DK-C61Q

[ref17] Duffy F, Sharpe H and Schwannauer M (2019) The effectiveness of interpersonal psychotherapy for adolescents with depression – a systematic review and meta-analysis. Child and Adolescent Mental Health 24, 307–317. 32677350 10.1111/camh.12342

[ref18] Eldridge S, Lancaster G, Campbell M, Thabane L, Hopewell S, Coleman C and Bond C (2016) Defining feasibility and pilot studies in preparation for randomised controlled trials: development of a conceptual framework. PLoS ONE 11, e0150205. 26978655 10.1371/journal.pone.0150205 PMC4792418

[ref19] Fazel M, Patel V, Thomas S and Tol W (2014) Mental health interventions in schools in low-income and middle-income countries. The Lancet. Psychiatry 1, 388–398. 26361001 10.1016/S2215-0366(14)70357-8

[ref20] Gale NK, Heath G, Cameron E, Rashid S and Redwood S (2013) Using the framework method for the analysis of qualitative data in multi-disciplinary health research. BMC Medical Research Methodology 13, 117. 24047204 10.1186/1471-2288-13-117 PMC3848812

[ref21] Greco G, Knight L, Ssekadde W, Namy S, Naker D and Devries K (2018) Economic evaluation of the good school toolkit: an intervention for reducing violence in primary schools in Uganda. BMJ Global Health 3, e000526. 10.1136/bmjgh-2017-000526 PMC5914895 29707243

[ref22] Gronholm PC, Nye E and Michelson D (2018) Stigma related to targeted school-based mental health interventions: a systematic review of qualitative evidence. Journal of Affective Disorders 240, 17–26. 30041074 10.1016/j.jad.2018.07.023

[ref23] Gunlicks-Stoessel M, Mufson L, Jekal A and Turner JB (2010) The impact of perceived interpersonal functioning on treatment for adolescent depression: IPT-A versus treatment as usual in school-based health clinics. Journal of Consulting and Clinical Psychology 78, 260–267. 20350036 10.1037/a0018935 PMC2853239

[ref24] Hall GCN, Ibaraki AY, Huang ER, Marti CN and Stice E (2016) A meta-analysis of cultural adaptations of psychological interventions. Behavior Therapy 47, 993–1014. 27993346 10.1016/j.beth.2016.09.005

[ref25] Heim E and Kohrt BA (2019) Cultural adaptation of scalable psychological interventions: a new conceptual framework. Clinical Psychology in Europe 1, 1–22.

[ref26] Hyde JS and Mezulis AH (2020) Gender differences in depression: biological, affective, cognitive, and sociocultural factors. Harvard Review of Psychiatry 28, 4–13. 31913978 10.1097/HRP.0000000000000230

[ref27] International Monetary Fund (2020) IMF data [Online]. Available at https://data.imf.org (Accessed 9th March 2022).

[ref28] Ivarsson T, Lidberg A and Gillberg C (1994) The Birleson Depression Self-Rating Scale (DSRS). Clinical evaluation in an adolescent inpatient population. Journal of Affective Disorders 32, 115–125. 7829763 10.1016/0165-0327(94)90069-8

[ref29] Johnson JG, Harris ES, Spitzer RL and Williams JBW (2002) The patient health questionnaire for adolescents: validation of an instrument for the assessment of mental disorders among adolescent primary care patients. Journal of Adolescent Health 30, 196–204. 10.1016/s1054-139x(01)00333-0 11869927

[ref30] Jordans M, Komproe IH, Tol W, Kohrt BA, Luitel NP, Macy RD and De Jong JT (2010) Evaluation of a classroom-based psychosocial intervention in conflict-affected Nepal: a cluster randomized controlled trial. The Journal of Child Psychology and Psychiatry 51, 818–826. 20102428 10.1111/j.1469-7610.2010.02209.x

[ref31] Jordans MJD, Kohrt BA, Luitel NP, Komproe IH and Lund C (2015) Accuracy of proactive case finding for mental disorders by community informants in Nepal. British Journal of Psychiatry 207, 501–506. 10.1192/bjp.bp.113.141077 PMC4664856 26450582

[ref32] Jordans MJD, Coetzee A, Steen HF, Koppenol-Gonzalez GV, Galayini H, Diab SY, Aisha SA and Kohrt BA (2021) Assessment of service provider competency for child and adolescent psychological treatments and psychosocial services in global mental health: evaluation of feasibility and reliability of the WeACT tool in Gaza, Palestine. Global Mental Health 8, e7. 34026237 10.1017/gmh.2021.6 PMC8127642

[ref33] Kohrt BA and Harper I (2008) Navigating diagnoses: understanding mind-body relations, mental health, and stigma in Nepal. Culture, Medicine and Psychiatry 32, 462. 18784989 10.1007/s11013-008-9110-6 PMC3869091

[ref34] Kohrt BA, Kunz RD, Koirala NR, Sharma VD and Nepal MK (2003) Validation of the Nepali version of the Beck Anxiety Inventory. Journal of the Institute of Medicine 25, 1–4.

[ref35] Kohrt BA, Jordans M, Tol W, Luitel NP, Maharjan SM and Upadhaya N (2011) Validation of cross-cultural child mental health and psychosocial research instruments: adapting the Depression Self-Rating Scale and Child PTSD Symptom Scale in Nepal. BMC Psychiatry 11, 127. 10.1186/1471-244X-11-127 PMC3162495 21816045

[ref36] Kohrt BA, Jordans MJD, Rai S, Shrestha P, Luitel NP, Ramaiya MK, Singla DR and Patel V (2015) Therapist competence in global mental health: development of the ENhancing Assessment of Common Therapeutic factors (ENACT) rating scale. Behaviour Research and Therapy 69, 11–21. 25847276 10.1016/j.brat.2015.03.009 PMC4686771

[ref37] Kohrt BA, Luitel NP, Acharya P and Jordans MJD (2016) Detection of depression in low resource settings: validation of the Patient Health Questionnaire (PHQ-9) and cultural concepts of distress in Nepal. BMC Psychiatry 16, 58. 26951403 10.1186/s12888-016-0768-y PMC4782581

[ref38] Kohrt BA, Ottman K, Panter-Brick C, Konner M and Patel V (2020) Why we heal: the evolution of psychological healing and implications for global mental health. Clinical Psychology Review 82, 101920. 33126037 10.1016/j.cpr.2020.101920

[ref39] Kola L, Kohrt BA, Hanlon C, Naslund JA, Sikander S, Balaji M, Benjet C, Cheung EYL, Eaton J, Gonsalves P, Hailemariam M, Luitel NP, Machado DB, Misganaw E, Omigbodun O, Roberts T, Salisbury TT, Shidhaye R, Sunkel C, Ugo V, Van Rensburg AJ, Gureje O, Pathare S, Saxena S, Thornicroft G and Patel V (2021) COVID-19 mental health impact and responses in low-income and middle-income countries: reimagining global mental health. The Lancet. Psychiatry 8, 535–550. 33639109 10.1016/S2215-0366(21)00025-0 PMC9764935

[ref40] Luitel NP, Jordans MJD, Adhikari A, Upadhaya N, Hanlon C, Lund C and Komproe IH (2015) Mental health care in Nepal: current situation and challenges for development of a district mental health care plan. Conflict and Health 9, 3. 10.1186/s13031-014-0030-5 PMC4331482 25694792

[ref41] Markowitz JC and Weissman M (2004) Interpersonal psychotherapy: principles and applications. World Psychiatry 3, 136–139. 16633477 PMC1414693

[ref42] Meffert SM, Neylan TC, Mcculloch CE, Blum K, Cohen CR, Bukusi EA, Verdeli H, Markowitz JC, Kahn JG, Bukusi D, Thirumurthy H, Rota G, Rota R, Oketch G, Opiyo E and Ongeri L (2021) Interpersonal psychotherapy delivered by nonspecialists for depression and posttraumatic stress disorder among Kenyan HIV-positive women affected by gender-based violence: randomized controlled trial. PLoS Medicine 18, e1003468. 33428625 10.1371/journal.pmed.1003468 PMC7799784

[ref43] Michelson D, Malik K, Parikh R, Weiss HA, Doyle AM, Bhat B, Sahu R, Chilhate B, Mathur S, Krishna M, Sharma R, Sudhir P, King M, Cuijpers P, Chorpita B, Fairburn CG and Patel V (2020) Effectiveness of a brief lay counsellor-delivered, problem-solving intervention for adolescent mental health problems in urban, low-income schools in India: a randomised controlled trial. The Lancet Child & Adolescent Health 4, 571–582. 32585185 10.1016/S2352-4642(20)30173-5 PMC7386943

[ref44] Ministry of Health and Population (MOHP) (2012) Nepal Adolescents and Youth Survey 2010/11. Kathmandu, Nepal.

[ref45] Ministry of Home Affairs (2015) Nepal earthquake 2072: situation update as of the 11th May. Kathmandu.

[ref46] Mokdad AH, Forouzanfar MH, Daoud F, Mokdad AA, El Bcheraoui C, Moradi-Lakeh M, Kyu HH, Barber RM, Wagner J, Cercy K, Kravitz H, Coggeshall M, Chew A, O'Rourke KF, Steiner C, Tuffaha M, Charara R, Al-Ghamdi EA, Adi Y, Afifi RA, Alahmadi H, Albuhairan F, Allen N, Almazroa M, Al-Nehmi AA, Alrayess Z, Arora M, Azzopardi P, Barroso C, Basulaiman M, Bhutta ZA and Al E (2016) Global burden of diseases, injuries, and risk factors for young people's health during 1990–2013: a systematic analysis for the global burden of disease study 2013. Lancet 387, 2383–2401. 27174305 10.1016/S0140-6736(16)00648-6

[ref47] Mufson L, Weissman MM, Moreau D and Garfinkel R (1999) Efficacy of interpersonal psychotherapy for depressed adolescents. Archives of General Psychiatry 56, 573–579. 10359475 10.1001/archpsyc.56.6.573

[ref48] Mufson L, Dorta KP, Wickramaratne P, Nomura Y, Olfson M and Weissman MM (2004 *a*) A randomized effectiveness trial of interpersonal psychotherapy for depressed adolescents. Archives of General Psychiatry 61, 577–584. 15184237 10.1001/archpsyc.61.6.577

[ref49] Mufson L, Gallagher T, Dorta KP and Young JF (2004 *b*) A group adaptation of interpersonal psychotherapy for depressed adolescents. American Journal of Psychotherapy 58, 220–237. 15373283 10.1176/appi.psychotherapy.2004.58.2.220

[ref50] Nakimuli-Mpungu E, Musisi S, Wamala K, Okello J, Ndyanabangi S, Birungi J, Nanfuka M, Etukoit M, Mayora C, Ssengooba F, Mojtabai R, Nachega JB, Harari O and Mills EJ (2020) Effectiveness and cost-effectiveness of group support psychotherapy delivered by trained lay health workers for depression treatment among people with HIV in Uganda: a cluster-randomised trial. The Lancet Global Health 8, e387–e398. 32035035 10.1016/S2214-109X(19)30548-0

[ref52] O'Shea G, Spence SH and Donovan CL (2015) Group versus individual interpersonal psychotherapy for depressed adolescents. Behavioural and Cognitive Psychotherapy 43, 1–19. 25600515 10.1017/S1352465814000216

[ref51] Orben A, Tomova L and Blakemore S-J (2020) The effects of social deprivation on adolescent development and mental health. The Lancet Child & Adolescent Health 4, 634–640. 32540024 10.1016/S2352-4642(20)30186-3 PMC7292584

[ref53] Panter-Brick C, Eggerman M, Gonzalez V and Safdar S (2009) Violence, suffering, and mental health in Afghanistan: a school-based survey. Lancet 374, 807–816. 19699514 10.1016/S0140-6736(09)61080-1 PMC2748901

[ref54] Patel V, Weiss HA, Chowdhary N, Naik S, Pednekar S, Chatterjee S, De Silva MJ, Bhat B, Araya R, King M, Simon G, Verdeli H and Kirkwood BR (2010) Effectiveness of an intervention led by lay health counsellors for depressive and anxiety disorders in primary care in Goa, India (MANAS): a cluster randomised controlled trial. Lancet 376, 2086–2095. 21159375 10.1016/S0140-6736(10)61508-5 PMC4964905

[ref55] Patel V, Saxena S, Lund C, Thornicroft G, Baingana F, Bolton P, Chisholm D, Collins PY, Cooper JL, Eaton J, Herrman H, Herzallah MM, Huang Y, Jordans MJD, Kleinman A, Medina-Mora ME, Morgan E, Niaz U, Omigbodun O, Prince M, Rahman A, Saraceno B, Sarkar BK, De Silva M, Singh I, Stein DJ, Sunkel C and Unützer J (2018) The lancet commission on global mental health and sustainable development. The Lancet 392, 1553–1598. 10.1016/S0140-6736(18)31612-X 30314863

[ref56] Petersen I, Bhana A and Baillie K Mhapp Research Programme Consortium (2012) The feasibility of adapted group-based interpersonal therapy (IPT) for the treatment of depression by community health workers within the context of task shifting in South Africa. Community Mental Health Journal 48, 336–341. 21687982 10.1007/s10597-011-9429-2

[ref57] Petersen I, Hanass Hancock J, Bhana A and Govender K (2014) A group-based counselling intervention for depression comorbid with HIV/AIDS using a task shifting approach in South Africa: a randomized controlled pilot study. Journal of Affective Disorders 158, 78–84. 24655769 10.1016/j.jad.2014.02.013

[ref58] Rice S, Oliffe J, Seidler Z, Borschmann R, Pirkis J, Reavley N and Patton G (2021) Gender norms and the mental health of boys and young men. The Lancet Public Health 6, e541–e542. 34332667 10.1016/S2468-2667(21)00138-9

[ref59] Rose-Clarke K, Pradhan I, Shrestha P, Prakash BK, Magar J, Luitel NP, Devakumar D, Rafaeli AK, Clougherty K, Kohrt BA, Jordans MJD and Verdeli H (2020) Culturally and developmentally adapting group interpersonal therapy for adolescents with depression in rural Nepal. BMC Psychology 8, 83. 10.1186/s40359-020-00452-y PMC7425581 32787932

[ref60] Rose-Clarke K, Hassan E, Bk P, Magar J, Devakumar D, Np L, Verdeli H and Ba K (2021) A cross-cultural interpersonal model of adolescent depression: a qualitative study in rural Nepal. Social Science & Medicine 270, 113623. 33461033 10.1016/j.socscimed.2020.113623 PMC7895817

[ref61] Rosselló J, Bernal G and Rivera-Medina C (2008) Individual and group CBT and IPT for Puerto Rican adolescents with depressive symptoms. Cultural Diversity & Ethnic Minority Psychology 14, 234–245. 18624588 10.1037/1099-9809.14.3.234

[ref62] Sangraula M, Kohrt BA, Ghimire R, Shrestha P, Luitel NP, Van't Hof E, Dawson K and Jordans MJD (2021) Development of the mental health cultural adaptation and contextualization for implementation (mhCACI) procedure: a systematic framework to prepare evidence-based psychological interventions for scaling. Global Mental Health 8, e6–e6. 33996110 10.1017/gmh.2021.5 PMC8082944

[ref63] Silwal S, Dybdahl R, Chudal R, Sourander A and Lien L (2018) Psychiatric symptoms experienced by adolescents in Nepal following the 2015 earthquakes. Journal of Affective Disorders 234, 239–246. 29549825 10.1016/j.jad.2018.03.002

[ref64] Swartz HA, Grote NK and Graham P (2014) Brief interpersonal psychotherapy (IPT-B): overview and review of evidence. American Journal of Psychotherapy 68, 443–462. 26453346 10.1176/appi.psychotherapy.2014.68.4.443 PMC4603530

[ref65] Tang T-C, Jou S-H, Ko C-H, Huang S-Y and Yen C-F (2009) Randomized study of school-based intensive interpersonal psychotherapy for depressed adolescents with suicidal risk and parasuicide behaviors. Psychiatry and Clinical Neurosciences 63, 463–470. 19531111 10.1111/j.1440-1819.2009.01991.x

[ref66] Thapar A, Collishaw S, Pine DS and Thapar AK (2012) Depression in adolescence. Lancet 379, 1056–1067. 22305766 10.1016/S0140-6736(11)60871-4 PMC3488279

[ref67] Tol W, Jordans M, Regmi S and Sharma B (2005) Cultural challenges to psychosocial counselling in Nepal. Transcultural Psychiatry 42, 317–333. 16114588 10.1177/1363461505052670

[ref68] Toth SL, Handley ED, Manly JT, Sturm R, Adams TR, Demeusy EM and Cicchetti D (2020) The moderating role of child maltreatment in treatment efficacy for adolescent depression. Journal of Abnormal Child Psychology 48, 1351–1365. 32696103 10.1007/s10802-020-00682-z PMC7484366

[ref70] UN Women (2016) United Nations Entity for Gender Equality and the Empowerment of Women. Sindhupalchok Gender Profile. Available at https://www.humanitarianresponse.info/en/node/130258 (Accessed 16th August 2022).

[ref69] UNICEF (2021) The State of the World's Children 2021: On My Mind – Promoting, protecting and caring for children's mental health. New York.

[ref71] Van Ginneken N, Chin WY, Lim YC, Ussif A, Singh R, Shahmalak U, Purgato M, Rojas-García A, Uphoff E, Mcmullen S, Foss HS, Thapa Pachya A, Rashidian L, Borghesani A, Henschke N, Chong LY and Lewin S (2021) Primary-level worker interventions for the care of people living with mental disorders and distress in low- and middle-income countries. The Cochrane Database of Systematic Reviews 8, Cd009149. 34352116 10.1002/14651858.CD009149.pub3 PMC8406740

[ref72] World Bank (2020) World Bank data [Online]. Available at https://data.worldbank.org/ (Accessed 9th March 2022).

[ref73] World Health Organization (2016) mhGAP Intervention Guide – Version 2.0 for mental, neurological and substance use disorders in non-specialised health settings. 27786430

[ref74] World Health Organization and Columbia University (2016) Group interpersonal therapy (IPT) for depression. (WHO generic field-trial version 1.0). Geneva: WHO.

[ref75] Zhou X, Hetrick SE, Cuijpers P, Qin B, Barth J, Whittington CJ, Cohen D, Del Giovane C, Liu Y, Michael KD, Zhang Y, Weisz JR and Xie P (2015) Comparative efficacy and acceptability of psychotherapies for depression in children and adolescents: a systematic review and network meta-analysis. World Psychiatry 14, 207–222. 26043339 10.1002/wps.20217 PMC4471978

[ref76] Zhou X, Teng T, Zhang Y, Del Giovane C, Furukawa TA, Weisz JR, Li X, Cuijpers P, Coghill D, Xiang Y, Hetrick SE, Leucht S, Qin M, Barth J, Ravindran AV, Yang L, Curry J, Fan L, Silva SG, Cipriani A and Xie P (2020) Comparative efficacy and acceptability of antidepressants, psychotherapies, and their combination for acute treatment of children and adolescents with depressive disorder: a systematic review and network meta-analysis. The Lancet. Psychiatry 7, 581–601. 32563306 10.1016/S2215-0366(20)30137-1 PMC7303954

