# Supplementary material for: School-based group interpersonal therapy for adolescents with depression in rural Nepal: a mixed methods study exploring feasibility, acceptability, and cost
Source: Glob Ment Health (Camb). 2022 Aug 22;9:416–28. doi: 10.1017/gmh.2022.46 (PMC9806967; doi:10.1017/gmh.2022.46)
Supplement: Supplementary file 1 [file S2054425122000462sup001.docx]

**SUPPLEMENTARY MATERIAL**

**Appendix A:** Baseline characteristics of adolescents by school

| **School**  **(groups)** | **School 1**  **(F1, F2, F3, M1)** | **School 2**  **(F4, M2)** | **School 3**  **(M4)** | **School 4**  **(M3)** |
| --- | --- | --- | --- | --- |
| N. students | 30 | 16 | 8 | 8 |
| % female | 73.3 | 50 | 0 | 0 |
| **Outcomes (average score)** | | | | |
| DSRS | 18.0 | 16.9 | 15.4 | 16.5 |
|  |  |  |  |  |
| Functional I. | 12.5 | 12.6 | 11.9 | 13.5 |
| PHQ | 10.8 | 9.3 | 9.8 | 8.8 |
| BAI | 18.2 | 16.1 | 13.8 | 14.9 |
| Child PTSD | 25.8 | 26.0 | 24.3 | 21.1 |
| Behavioural index | 7 | 3.8 | 7 | 7.1 |
| **Covariates (average %)** | | |  |  |
| Age (mean, SD) | 15.2 (1.4) | 15.3 (0.9) | 14.3 (0.9) | 14.1 (1.0) |
| Caste: Brah/Chh | 40.0 (n = 12) | 50.0 (n = 8) | 37.5 (n = 3) | 37.5 (n = 3) |
| Janajati | 56.7 (n = 17) | 43.8 (n = 7) | 62.5 (n = 5) | 50.0 (n = 4) |
| Dalit | 3.3 (n = 1) | 6.3 (n = 1) | 0.0 (n = 0) | 12.5 (n = 1) |
| Low income* | 63.3 (n = 19) | 93.8 (n = 15) | 50.0 (n = 4) | 62.5 (n = 5) |
| Nuclear family | 50.0 (n = 15) | 56.3 (n = 9) | 75.0 (n = 6) | 87.5 (n = 7) |
| Live w/ parents | 63.3 (n = 19) | 68.8 (n = 11) | 50.0 (n = 4) | 87.5 (n = 7) |

* Low income = family income sufficient for no more than 6 months

**Appendix B:** Participant flowchart

***Appendix C:*** *Outcomes by wave and baseline age-group (13-14y vs 15-19y)*

**Appendix D:** Outcomes by wave and number of mental health comorbidities (> cutoff score on the DSRS + BAI/CPSS = 2 comorbidities, or DSRS + BAI + CPSS = 3 comorbidities)

**Appendix E:** Annual cost, unit cost and percentage cost related to start-up, implementation or intervention activity for the pilot study and if operating at capacity

|  | **Pilot study (n=62)** | **Full capacity (n=360)** |
| --- | --- | --- |
| **Average annual cost** | NPR 3,341,424  (Int’l $99,655; US $27,728) | NPR 4,055,368  (Int’l $120,947; US $34,867) |
| **Unit cost** | NPR 53,894  (Int’l $1,607; US $447) | NPR 11,265  (Int’l $336.0;  US $96.9) |
| **Start-up** | 30.2% | 26.9% |
| **Implementation** | 69.8% | 73.1% |
| **IPT facilitation** | 28.6% | 40.4% |
| **Training and recruitment** | 29.0% | 23.4% |
| **Screening and referral** | 10.8% | 9.1% |
| **Adaptation** | 1.1% | 0.4% |
| **Monitoring & evaluation** | 30.5% | 26.8% |
